# Innovative Concepts and Recent Breakthrough for Engineered Graft and Constructs for Bone Regeneration: A Literature Systematic Review

**DOI:** 10.3390/ma15031120

**Published:** 2022-01-31

**Authors:** Francesco Inchingolo, Denisa Hazballa, Alessio Danilo Inchingolo, Giuseppina Malcangi, Grazia Marinelli, Antonio Mancini, Maria Elena Maggiore, Ioana Roxana Bordea, Antonio Scarano, Marco Farronato, Gianluca Martino Tartaglia, Felice Lorusso, Angelo Michele Inchingolo, Gianna Dipalma

**Affiliations:** 1 Department of Interdisciplinary Medicine, University of Medicine Aldo Moro, 70124 Bari, Italy; denisahazballa@gmail.com (D.H.); ad.inchingolo@libero.it (A.D.I.); giuseppinamalcangi@libero.it (G.M.); graziamarinelli@live.it (G.M.); dr.antonio.mancini@gmail.com (A.M.); m.e_maggiore@yahoo.it (M.E.M.); angeloinchingolo@gmail.com (A.M.I.); 2 Kongresi Elbasanit, Rruga: Aqif Pasha, 3001 Elbasan, Albania; 3 Department of Oral Rehabilitation, Faculty of Dentistry, Iuliu Hațieganu University of Medicine and Pharmacy, 400012 Cluj-Napoca, Romania; roxana.bordea@ymail.com; 4 Department of Innovative Technologies in Medicine and Dentistry, University of Chieti-Pescara, 66100 Chieti, Italy; ascarano@unich.it; 5 UOC Maxillo-Facial Surgery and Dentistry, Department of Biomedical, Surgical and Dental Sciences, School of Dentistry, Fondazione IRCCS Ca Granda, Ospedale Maggiore Policlinico, University of Milan, 20100 Milan, Italy; marco.farronato@unimi.it (M.F.); gianluca.tartaglia@unimi.it (G.M.T.)

**Keywords:** autologous teeth bone substitute, platelet derivates growth factors, PRP, PRF, CGF, APAG, low level laser therapy, graft, bone substitutes, bio-ceramics, scaffold

## Abstract

Background: For decades, regenerative medicine and dentistry have been improved with new therapies and innovative clinical protocols. The aim of the present investigation was to evaluate through a critical review the recent innovations in the field of bone regeneration with a focus on the healing potentials and clinical protocols of bone substitutes combined with engineered constructs, growth factors and photobiomodulation applications. Methods: A Boolean systematic search was conducted by PubMed/Medline, PubMed/Central, Web of Science and Google scholar databases according to the PRISMA guidelines. Results: After the initial screening, a total of 304 papers were considered eligible for the qualitative synthesis. The articles included were categorized according to the main topics: alloplastic bone substitutes, autologous teeth derived substitutes, xenografts, platelet-derived concentrates, laser therapy, microbiota and bone metabolism and mesenchymal cells construct. Conclusions: The effectiveness of the present investigation showed that the use of biocompatible and bio-resorbable bone substitutes are related to the high-predictability of the bone regeneration protocols, while the oral microbiota and systemic health of the patient produce a clinical advantage for the long-term success of the regeneration procedures and implant-supported restorations. The use of growth factors is able to reduce the co-morbidity of the regenerative procedure ameliorating the post-operative healing phase. The LLLT is an adjuvant protocol to improve the soft and hard tissues response for bone regeneration treatment protocols.

## 1. Introduction

In recent years, the bone regeneration procedure for implant-supported rehabilitations gained increased predictability due to innovative biomaterials, new generation bone grafts and substitutes and novel adjuvant therapies able to increase the osseointegration, the new bone formation and substitution, promote the bone remodeling and decrease the post-operative co-mobility and healing period. In fact, the objective of bone regeneration protocols is determined by the long-term predictability and survival rate of the implant rehabilitation and the control of the patient’s general and local risks factors [1,2,3,4,5,6,7,8,9,10,11]. In terms of a general clinical evaluation of the patient, the presence of systemic disorders, neoplasms, chemotherapy and radiotherapies, the health of the gastrointestinal tract, systemic pH balance and the local release of metallic ions related to implant positioning could determine a considerable influence on the immune/metabolic system [12,13]. Moreover, the autologous tooth-derived graft materialinistration of drugs acting on vascularization and bone metabolism and the evidence of chronic infections could determine an increased risk of implant failure [14,15,16]. A recent review that evaluated a total of 120 clinical and radiographical prospective studies stated that the implant survival rate was 85% after 1 year and 78% after 10 years with a success rate of 81%. Age-related degenerative and periodontal diseases were visibly connected to a general degenerative metabolic state in conjunction with systemic dysbiosis involving the oral cavity and the intestinal compartment. These conditions represent the cause of an increased degree of loss and wear of both implants and prostheses. Moreover, the peri-implantitis could be found in patients with low standards of both oral hygiene and a high degree of dysbiosis with a high bacterial load and chronic inflammation, even in patients with a regular follow-up schedule. Dysbiosis has been associated with a pseudo-allergic state with alterations of immune responses and genes polymorphisms (SNPs) that regulate the expression of immuno-modulating and pro-inflammatory responses: IL-10, IL-6 TNF, IFN IL-1 and VDR (vitamin D metabolism). These alterations have been mainly observed in patients with implant rehabilitations with at least three or more units and also in patients with metabolic, cardiovascular, renal deficiency and diabetes comorbidities [17,18,19,20]. The need to develop new, more efficient materials and techniques for functional and aesthetic rehabilitations remains a priority for researchers in the dental field [21,22,23,24,25,26,27]. A biomaterial that is used as a bone substitute should possess certain qualitative criteria: biocompatibility, which represents the capability of providing osseointegration without causing inflammatory reactions [28,29,30,31,32], osteoconductivity, the natural properties that allow cell activity, reproduction and amplification, and at last osteoinductive properties, or to be capable of triggering the bio-chemical and modulating processes, so stem cells can differentiate into osteoblasts, osteoclasts and osteocytes and induce osteogenesis, which is related to the formation of a new bone matrix [1,33,34,35]. Moreover, osteoinductive biomaterials should be able to recruit progenitor cells (MCS) to the grafted site, induce the formation of osteoblasts by differentiating progenitor cells (MCS) in mature cells and eventually regenerate ectopic bone where there is no extraskeletal structure [36]. The prevalence of implant failure and wear has been related to the presence of inflammation and infections as a result of oral diseases, such as peri-implant and oral flora disorders/dysbiosis [37,38,39,40,41,42,43,44,45,46,47]. The biophysical and biochemical properties of biomaterials could influence the cellular responses, including the macro-topography, pore geometry and stiffness, surface chemistry, rate of degradation and presence of biomolecules, influence proliferation and differentiation to eventually give tissue regeneration [48,49]. Healing of bone tissue occurs as a result of coupling activity between osteoclasts and osteoblasts and stem cells with immune cells. The inflammatory response of the host body is important for activating biochemical signals that bring the immune cells to the implant region, although chronic inflammation may be the cause of the implant failure [50]. The materials used in bone regeneration are divided into four main groups according to the origin of the biomaterials: alloplastic grafts, autologous grafts, xenografts and allografts [51].

These bone substitutes are biocompatible and bioabsorbable, able to induce the formation of new bone and maintain the bone volume. However, the biocompatibility and the bioabsorbable/preservation ratio of bone volume need to be improved, through research, to obtain increasingly predictable and favorable results for rapid bone regeneration [52]. The aim of the present investigation was to evaluate through a critical systematic overview of the recent innovations in the field of bone regeneration of alloplastic bone substitutes, peripheral mesenchymal cells constructs, growth factors and photobiomodulation applications. The aim of the present review was to perform a systematic review of the recent innovations in the field of bone regeneration with a focus on the healing potentials and clinical protocols of bone substitutes combined with engineered constructs, growth factors and photobiomodulation applications.

## 2. Materials and Methods

### 2.1. Article Search Methodology

The present systematic search was conducted by PubMed/Medline, PubMed/Central, Web of Science and Google scholar according to the PRISMA guidelines [53]. The article search was performed according to an ad hoc combination of Boolean operators (AND, OR) with the keywords on the topics of the present review. The keyword searchline is presented in Table 1.

### 2.2. Inclusion Criteria

For the present systematic search, the inclusion criteria were in vivo articles on human and animal studies in the field of cranio-maxillofacial bone regeneration that highlighted the characteristics of engineered bone constructs and combinations of growth factors and photobiomodulation applications.

### 2.3. Exclusion Criteria

The exclusion criteria considered for the descriptive analysis were, letter to the editor, articles written in non-English languages.

### 2.4. Paper Selection Process

The eligibility of the manuscripts for the descriptive review was performed independently by two reviewers evaluating the manuscript title and abstract. The full text was obtained and evaluated in this first stage if the abstract was unavailable. The papers not in line with the inclusion criteria were excluded from the analysis. The full text of the included manuscripts was collected and evaluated. The excluded articles were categorized according to the reasons for exclusion.

## 3. Results and Discussion

### General Parameters

**Figure 1:**
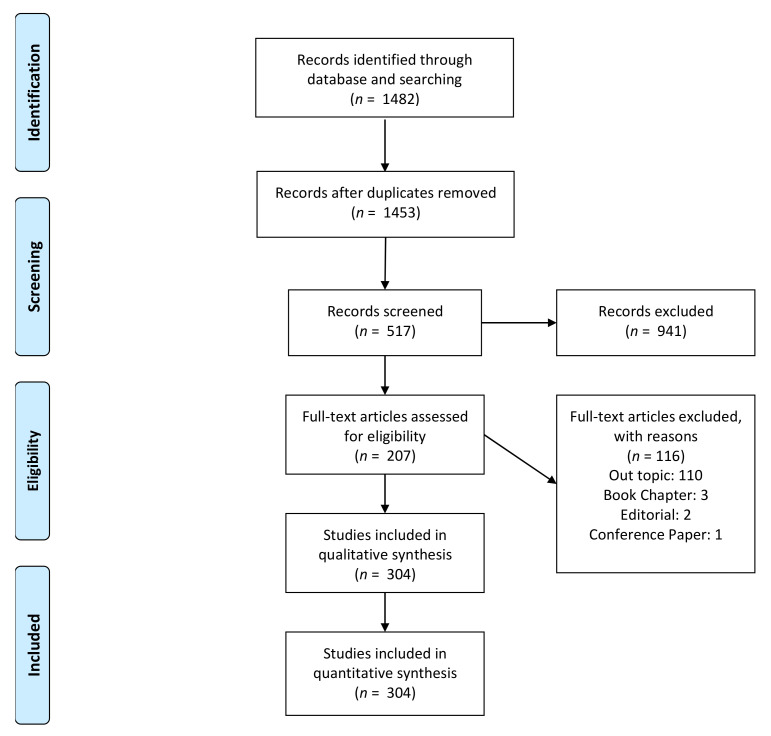
PRISMA flowchart of the included articles.

A total of 1448 articles were identified after the initial screening and a total of 304 papers were included for the qualitative synthesis (Figure 1).

## 4. Bone Substitutes and Graft

### 4.1. Alloplastic Grafts

Alloplastic bone grafts are synthetic biomaterials with no risks of antigenic reactions or transmission of various diseases. Many materials have been proposed as bone grafts containing calcium phosphate derivates with high biocompatibility. Calcium phosphate is similar to the inorganic components of human bones [54]. Hydroxyapatite (HA) Ca_10_(PO_4_)_6_(OH)_2_ is present in the human body, bones and teeth as an inorganic mineral, but in addition to the inorganic matrix; a consistent component of the human bone is the organic part that consists of type 1 proteins and collagen [55]. In a physiological environment, HA is not soluble; therefore, the formation of new bone occurs only through the reabsorption of these materials. The osteoclast activity is not optimal against the biphasic calcium phosphate (BCP) in 75% HA and 25% β-TCP. The solubility also influences the osteoclastic resorption pattern. The β-TCP has the highest solubility and HA the lowest, so the osteoclasts’ reabsorption of the biomaterial proceeded in the right proportion to the solubility of the substances, but this is not entirely accurate [56]. Zwingenberger et al. reported similarly to Yamada et al. that HA with HA/β-TCP 75/25% mix is not completely reabsorbed, but β-TCP has a higher solubility than HA/β-TPC 25/75% but HA/β-TPC 25/75% had better osteoclastic resorption [56,57]. Ortiz-Puigpelat et al. reported that HA/TCP 50% ratio is more appropriate in bone regeneration for the percentage of residual material and new newly formed bone after 12–24 weeks [58]. The hydroxyapatite (HA) and calcium tri-phosphate (TPC) are characterized by high compressive strength (statical forces) but are very fragile due to dynamic forces, so this feature limits their use in large bone defects [59]. In operative clinical practice, these defects are correlated to various causes, such as bone tumors, severe trauma or drugs abuse with an alteration in bone metabolism. In a pure form, HA and β-TCP have been widely used as biodegradable coatings. HA and β-TCP also prevent the formation of fibrotic tissue around the implants derived by the body’s immune response. This fibrotic tissue can also lead to implant failure [60,61]. The porosity of calcium phosphates is one of the factors that induce their poor initial mechanical property, but the porosity is able to influence the early fibro-vascularization of the graft and replace it with new bone [62]. Moreover, the scaffold pore size is able to affect the progression of osteogenesis. Small pores promote hypoxic conditions and induce osteochondral formation before osteogenesis occurs. On the other hand, larger scaffold pores allow vascularization, directly evoking the onset of osteogenesis [63,64]. The optimal pore diameter of bone substitutes should be between 0.2 and 0.5 mm [65,66], while Ghayer and Weber reported an optimal pore range between 0.7 and 1.2 mm with excellent results in healing cranial defects [67]. Another factor of these biomaterials for the new bone formation is the presence of micropores [68], while a submicron micropore structure <10 µm has been reported to accelerate the formation of new bone, and the quantity of these micropores improves the results [69]. Many studies are focused on compound forms or biphasic forms, thus improving the mechanical properties, solubility and integration of these biomaterials [59,60,70,71,72,73]. De Tullio et al. used bone replacement materials, such as calcium sulphate (CS) and nano-hydroxyapatite (NHA), in a comparative study to prevent a reduction in the alveolar ridge after tooth extraction [74]. After 5 months, the histomorphometric analysis showed that these biomaterials led to bone formation, but the combination of CS with NHA showed better results in the regeneration of new bone and in the number of residual graft particles [74]. Lin et al. reported on 51 patients that the HA/TCP+ collagen composite gave excellent results in preserving post-extraction alveolar bone [75]. Maji et al. in order to improve the mechanical properties, suggested gelatin-chitosan-β-TCP 30% with high porosity the GCT 30 scaffold. This scaffold has improved the mechanical properties showing high strength and compressive strength of the new bone up to 2.5 MPa, which is also the lower range of cancellous bone. The change in the percentage of β-TCP changes the absorption of proteins, and GCT 30 also has the ability to stimulate mesenchymal stem cells (MSCs) and transform them into osteoblasts, thus stimulating angiogenesis [76]. Afroze et al. proposed a different combination of HA with the addition of a f_MWCNT_ 0.5 wt% multi-walled carbon nanotube. The study was carried out in vitro with the aim of improving the mechanical properties of HA without modifying its structure. In fact, the HA, which contains carbon, seems to have greater similarity with the inorganic phase of the bone [77]. The fMWCNT prevents the HA from decomposing into α-TCP and Ca2P2O7, and it promotes the porosity of the composite, which is essential for the growth of cells. This combination of HA with 0.5 wt% low dose fMWCNT is favorable for improving mechanical strength by approximately 317.5%, hardness by 172.4% and fracture strength by 237% [77]. This nanocomposite’s favorable application, as has been demonstrated, is when the bone will be a load-bearing support for implants [77]. Bone materials, such as β-TCP, have the ability to induce bone regeneration through the release of Ca ions [78]. Synthetic materials have high osteoconductive properties but no evidence of osteoinductivity [79]. Recently, a new synthetic biomaterial named Osopia has been suggested, in relation of its osteoinductive properties. This biomaterial is composed of the combination of HA and β-TCP to obtain medium resorption properties. The structure of the Osopia is extended in macropores with a submicronic surface. The submicronic surface structure promotes osteogenesis by regulating the transforming growth factor (β-TGF) pathway and thus inducing the differentiation of mesenchymal stem cell (MSC) [80]. The topographic/chemical characteristic of this biomaterial drives self-induction and consequently the differentiation of osteoblasts [80]. Chen et al. confirmed the possibility that a biphasic HA/TCP can induce ectopic bone in non-osseous sites and that this ability is closely related to the immune response that is caused by this material [81]. A new bio-scaffold called Compact Bio Bone Cells has recently been tested. Since this scaffold is composed of β-TCP, fibrin gel matrix and peripheral blood stem cells, it creates an imitation of the microenvironment of the bone tissue. The in vitro study showed that the Compact Bio Bone Cells had a comparable biodegradability rate to the bone regeneration rate, so at 7–12 days, cells resembling mature osteoblasts were highlighted. The presence of stem cells induces the vascularization of the region [82]. Even when stem cells are harvested from the pulp of the tooth, it is suggested to use them in bone regeneration. In combination with synthetic scaffolds, stem cells originating from the dental pulp have given excellent results in tissue neoformation in a shorter time [83]. The combination of TCP with platelet-rich plasma (PRF) has also been suggested in the case of fractures. This combination not only provides bone regeneration (resorption occurs at the same time as bone regeneration), but the presence of PRF aids in the healing of the positioning soft tissues [84].

The recent advances in reconstructive dentistry are oriented towards using bioactive glasses due to the increased potential bioactivity and biocompatibility for bone regeneration procedures [85]. Recent studies reported that the biocompatibility of these biomaterials is correlated with the silicate-derived percentage range between 45 and 52%, which represents an optimal equilibrium between the tissue tolerance and the osteogenesis capability of the graft [85,86,87,88,89]. The bioglasses-acting mechanism is produced by the local release of ions and the generation of amorphous calcium phosphates precipitates that are able to generate a local change of the pH levels and osmotic flows [90,91,92,93]. Due to the new bone formation properties, the bioglasses are able to interact with the grafting environment and modulate the activity of several osteogenetic factors, such as the alkaline phosphatase and bone morphogenetic proteins (BMP2).

### 4.2. Autologous Graft

**Figure 2:**
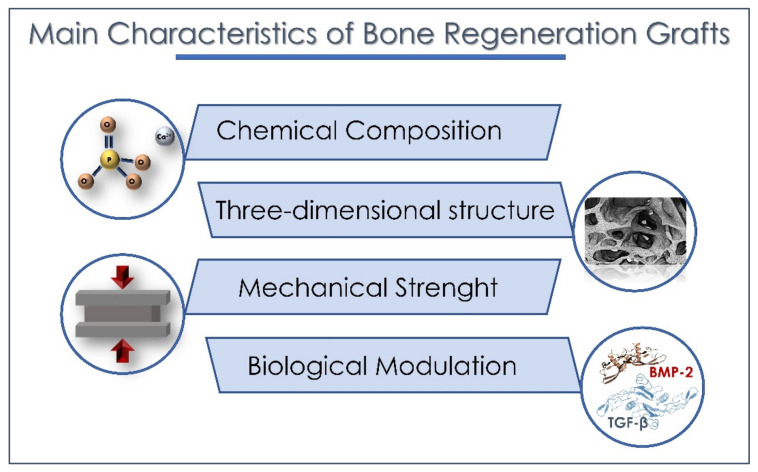
Synthesis of the biomaterials for bone regeneration.

The dental matrix has also been tested and used for a long time as a bone regeneration material [94,95]. After extraction, the tooth is considered a useless material and is eliminated, and for this reason, the use of this material has a low cost. The dental matrix contains HA and type 1, type 3 and type 5 collagen, and proteins are very similar to alveolar bone as they are both derived from the cells of the neural crest [55,96,97,98]. The hydroxyapatite present in the dental matrix has the same composition of HA as that present in bone, and for this reason, it is similar to the synthetic one [99]. The osteoconductive properties of tooth-derived materials are widely accepted by several authors [96,100,101]. In fact, the tooth can be considered the best natural scaffold. Comparing the dental matrix to HA/TCP, it is shown to be more biocompatible and bioactive [98,102]. Dentin is composed of inorganic material (65%) and organic matrix and water (35%). The inorganic part, HA [3Ca_3_ (PO4) 2Ca (OH)_2_], has crystals 10-times larger than those of bone (this is the biggest difference between these materials) and 300-times smaller than those of enamel [98,103]. The organic part is composed of 90% type 1 collagen and 10% non-collagenic proteins including growth factors (BMPs) and enzymes, such as alkalin and acid phosphatases and MMPs [98,103]. The presence of growth factors (GFs), as well as mesenchymal cells, is also found in the apical part of the wisdom teeth [104] and in the dental pulp in a considerable amount [105]. In 1965, Marshall R. Urist hypothesized the presence of proteins (BMP) in the bone matrix, and these proteins have the ability to induce ectopic bone [106,107]. BMPs play an important role in the differentiation of mesenchymal cells into osteoblasts [108], in addition to stimulating angiogenesis [109,110]. In 1991, Bessho et al. observed the presence of the bone morphogenic protein (BMP) in the dentin matrix [111]. Many researchers have focused on (BMPs) to evaluate the effectiveness of this low-molecular-weight glycoprotein [98,108,109,110,111,112,113,114,115]. In the BMP family, BMP-9 is considered the protein with the highest osteogenic potential. BMP-9 is capable of inducing the formation of new bone in critical defects by intervening in the differentiation of progenitor cells and in angiogenesis [110,112]. BMP-2 is also one of the strongest stimulants among growth factors. Its use has led to results comparable to those of autologous grafts in terms of bone volume and density and even reduced the risk of infection and the time of hospitalization [113]. It has been reported that bone repair could be produced by BMP-2, but this ability is dose-dependent [114], while a high dosage could give unwanted effects [115,116]. In the tooth matrix, the BMP is found in physiological quantities and therefore adequate for the patient. The discussion continues on how this material will be used—mineralized or demineralized. The tooth crushing technique is a tooth processing method that is proposed for use as an autologous bone graft. This technique was initially applied to the newly extracted wisdom tooth. The tooth was crushed with a bone mill and hammer without being made into a solution in order to retain the pulp. Six months after the surgery, radiological and clinical control showed that there was normal bone healing [117]. Other researchers also support the techniques with the tooth not transformed with acids, aiming to preserve the osteoinductive properties [118,119]. On the other hand, numerous studies evaluate the demineralized and granulated material as a bone graft. Woong et al. state that the demineralized dentin acts as a carrier for the BMP-2 contained within the dental structure, thus combining the properties of the scaffold with the cells of the BMP-2 [120]. After being treated with acids, the dentin releases Ca^2+^ ions, thus creating a porous graft that acts as a perfect scaffold. The porous structure will contribute not only to the circulation of proteins (BMP) but also to the positioning of the fibrovascular network, which will help osteogenesis [121]. Rijal et al. compared the mineralized tooth matrix (MDM) as a bone graft with the demineralized tooth matrix (DDM). Human teeth were treated, and the experiment was performed on rats. DDM gave positive results, allowing the formation of new bone, thus demonstrating the regenerative abilities of this autologous material. This study did not confirm the same for MDM; in fact, the mineralized tooth matrix fails to trigger the regeneration of new bone and takes longer to re-absorb. The authors express the need for a protocol for the demineralization of the tooth so that this material can maintain its osteoinductive and osteoconductive properties [121]. In a recent review of the dental graft by Gharupure and Bhatavadekar, they analyzed all the different protocols for using a tooth as a graft material and found that new bone was created around the graft material, but the dental bone graft was not fully absorbed and replaced with new bone. The amount of residual non-resorbed dental bone graft is approximately equal to that of bovine bone [122]. This study found a significant number of complications, such as the separation of the mineralized block of the scaffold in some cases, causing the loss of primary stability to the implant, lack of osseointegration in others and loss of marginal bone more than one millimeter after 5 years showed an increased risk of dehiscence. As many procedures are empirical and not really standardized, this leads the authors to conclude that a standardization is needed for the procedure of transforming the tooth into microparticles that will be used as a graft [122]. During the demineralization process, the dentinal tubules will turn into channels for the circulation and the release of proteins as they will become wider. The protocol used for the demineralization of the tooth is very important as it can also lead to the complete destruction of dentinal tubules [102]. Further, the technique uses six liquids in addition to a partially controlled demineralization. With this patent method, the disinfection of the dental matrix is allowed, giving, in the end, a graft of 0.4–0.8 mm, which is easy to handle and apply [98,123]. Minetti et al. evaluated the Tooth Transformer (TT), which performs the transformation of the tooth by also disinfecting the dental matrix. This procedure lasts 25 min and can be performed in the same session immediately after tooth avulsion. It is totally automatic, thus reducing the risk of human error. Koga et al. state that if the dental matrix is partially demineralized with particles around 500–1000 µm, it has greater regeneration potential than when it has been completely demineralized, as it preserves more growth factors that will intervene in osteogenesis [124]. Bono et al. Support the idea of using treated and demineralized teeth, confirming that the demineralization performed with TT increases the bioavailability of BMP-2. The protocol used includes six solutions that demineralize and sterilize the crushed tooth [125]. It turns out that they do not damage the microstructure of dentin and organic matrix, as has been stated in other studies [126,127], but they increase the availability of BMP-2 in the matrix [98,125]. Substances that determine too high a demineralization (substances that give a high demineralization) produce a graft material of dental origin with low osteogenic potential as they eliminate or decrease the proteins present in the tooth, including BMP-2 [98]. Bono et al. affirm that this slight demineralization of the tooth lowers (reduces) the content of Ca and P compared to the non-demineralized matrix, but at the same time, it increases the bioavailability of BMP-2 [125,128]. BMP-2 increases the activity of ALP (alkaline phosphatase), which leads to an increase in osteodifferentiation. To induce significant ALP activity, a minimum concentration of 12.5 ng/mL of BMP-2 is needed, and after treatment with TT, the concentration of BMP-2 in the dentin matrix is 22 ng/mL [98,128]. An evaluation through scanning electron microscopy has shown that the density, roughness and homogeneity of the autologous dental graft is relatively similar to that of autogenous cortical bone, with a surface containing both organic and mineralized material [129]. The technique with the autologous tooth elaborated with the Tooth Transformer has been tested in the alveolar socket preservation of the bone after the extraction of the teeth [98]. The authors state that the success of the implant after bone regeneration was 99.1%, and the time it took for bone healing to be ready for implant was four months. The demineralization and decontamination performed by the solution does not eliminate BMP-2 and collagen [130,131]. The same author continued his research by using the Tooth Transformer on sinus lift with the same technique on a demineralized and granulated tooth [98]. In this case, the implants were inserted after six months. In this study, no inflammatory reaction was observed around the graft, and the tooth material used as graft was perfectly integrated with the host tissue. Unlike other autologous grafts, the tooth matrix does not lose bone volume with the passage of months [132]. In large bone defects, there is the problem of the amount of grafting that is needed in these cases. Umebayashi et al. have suggested the use of autologous bone when the quantity of teeth is limited, which can also be mixed with other biomaterials [133]. The matrix of the autologous tooth was partially demineralized (APDDM) and used in combination with particulate cancellous and medullary bone (PCBM) in bilateral sinus lift and in anterior maxillary reconstruction. This combination showed the osteoinductive capacities of PBCM and the osteoconductivity of APDDM. The implant was performed after 3.5 months [133]. Another suggested combination was that with xenographic material (Bio-Oss), which gave positive results in bone regeneration, but compared with the dental matrix alone, this last one forms a greater amount of bone [134]. In any case, the dental matrix can be combined with other biomaterials in large bone defects, which provides very positive results [133,134,135,136]. From the physio-chemical side, the tooth can be considered a material similar to bone but with a higher mineralization. A completely demineralized or a mineralized tooth did not bring good results in bone regeneration. In fact, the decalcification serves to make the dentin more accessible to collagenolytic enzymes and also for the fact that demineralized surfaces are the most adherent for osteoblasts [98]. The use of Tooth Transformer is highly manageable and is totally automatic, with no risk of human error. The use of this machine offers shredding at low speed that allows for homogenous particles and more material to use. The protocol used here is the one that offers a ratio between Ca and P (1.70) that is closer to the natural ratio found in bone (1.67) and, moreover, preserves proteins in the dental matrix (Figure 2) [98].

### 4.3. Xenografts Bone Substitutes

Xenotransplantation or bone derived from another species has been applied for many years as regenerative bone material. Their component is hydroxyapatite. Bone hydroxyapatite (BHA) is obtained naturally and has a lower cost than synthetic hydroxyapatite. The presence of ions, such as Na+ and CO_3_, seems to influence the molecular structure with lower porosity and thicker microstructure [137]. It has been shown that for bovine bone, the best results are obtained when it is used in particles with a diameter of 1.0–2.0 mm. These dimensions provide the best results in preserving bone volume compared to micro-sized particles [138]. The same result was accepted by Kon et al. for autologous bone, where larger particles (1.0–2.0 mm) give the best result in the formation of new bone and small particles tend to be absorbed more quickly [139]. Bovine-derived xenograft is one such material, which has been recognized as biocompatible with other animal and human organisms [140]. This material acts as a scaffold and promotes osteoblastic activity [141]. The microporous structure of bovine bone, which is considered similar to that of cancellous bone, allows this material to have osteoconductive properties [142]. The researchers demonstrated bone healing when they examined the bovine bone, and it was found that the bone is compact and mineralized and surrounds the bovine graft particles. The colonization of capillaries and newly formed cells is appreciated in Haversian canals [140,141,142]. Piattelli et al. used an inorganic bovine bone Bio-Oss for a maxillary sinus lift. After six months, the histomorphometric analysis showed that 30% of the samples selected for biopsy that were removed with a 4 mm drill under saline irrigation to a depth of 10 mm and examined by microscopy still contained Bio-Oss particles, but these particles were also observed after four years. The authors’ autologous tooth-derived graft material behaves like a scaffold an implant but has a very slow resorption [143]. However, other authors think that the slow reabsorption of this material serves as a source of mineralization [144,145]. In 2005, Sanchez et al. tested a combination of platelet-rich plasma (PRP) on dogs. In this study, they stated that the use of PRP in these animals did not give significant changes in bone regeneration [146]. More recent research has continued to combine Bio-Oss with platelet-rich fibrin PRF [147,148,149]. This combination is used in sinus lift, and the implant site was ready in 106 days, less than four months after surgery. The authors declare 100% success in bone regeneration following the Choukroun protocol [147]. The PRF has a dense and strong fibrin matrix, which is a great advantage over PRP. The PRP has also failed in other studies [150,151,152], but it should also be mentioned that the denomination of blood-derived products has often been confusing, but lately, everything has already been clarified, and their classification is made based on architecture of fibrin and cellular content [147,150,151,153,154,155,156,157,158,159,160]. Xenografts with CGF Growth Factor Concentrate is another suggested method. The authors used the Silfradent device to allow the CGF to penetrate the bone graft (to better allow fusion). Their technique in the augmentation of atrophic ridges gave excellent results; in just four months, mineralized bone was evident and ready for the implant procedure [161,162]. Continuing to try and improve osteoconductive properties, another study suggests combining Bio-Oss with BMP-2. To increase the building activity of this protein, rhBMP/Bio-Oss was coated with heparinized dopamine. The result showed that new bone formation began four weeks after surgery, while in week eight, there was a huge difference in the rate of bone formation between this study group and the group that was only used in the Bio-Oss graft [163]. Another combination tested is that between bovine bone Bio-Oss and autologous bone, but these two materials have differences in the time of absorption and have often not given good results in bone regeneration [164]. As xenografts are available in significant quantities, there are many studies focusing on these materials. A recent review on the use of bovine bone highlighted in the conclusions that 58% of cases failed in osseointegration, while 83% of these continued to have pain even after a long time. The main factor causing this complication is the body’s immune response to this material [165]. Therefore, for xenografts, the main antigen is the epitop alpha Gal, and to inhibit this antigen, the xenograft should be decellularized, and only then can it be used as a scaffold. However, the risk of an antigenic reaction will always be there, especially for bovine bone [165]. A new combination of bovine bone with type 1 atelocollagen has been recommended to minimize the antigenic reaction. Indeed, this combination is regarded as a new generation of xenografts. Atelocollagen has a low risk of antigenic reaction and inhibits bacterial activity. Additionally, unlike most xenografts, this combination of bovine bone graft with atelocollagen is totally resorbable [80]. Despite the treatment that xenograft undergoes, in 2007, Terry et al. highlighted the first case of bovine spongiform H-type encephalopathy related to bone reconstruction with bovine bone [166]. This means that the risk of transmitting diseases from one species to another exists.

## 5. Platelet Derivates and Growth Factors

From the peripheral blood, a platelet-rich autologous concentrate is collected, which is considered a “good healer” obtained in a non-invasive and rapid way [167], and when combined with other biomaterials, gives excellent results [168,169]. The first piastin concentrate has been called PRP and normally contains 0.5 × 10^11^ platelets for each unit. PRP does not have good mechanical properties. Before being activated, PRP is in liquid form [170]. This platelet concentrate releases growth factors and cytokines in a very short time and then reabsorbs in about 12–14 days. PRP has given the best results in combination with an autogenous graft compared to other materials used in bone regeneration [171]. However, the PRP gel in periodontology and dentoalveolar surgery has lost its use with the discovery of PRF, which has a lower cost and is easier to use [155]. The second-generation PRF encapsulates leukocytes, cytokines and, above all, growth factors in an autologous fibrin matrix. This PRF has been produced in the absence of anticoagulants, and for this reason, it is considered 100% autologous material that facilitates tissue regeneration by eliminating the transmission of diseases through the blood [172]. The fibrin matrix will act as a 3D scaffold in which cells and proteins will be captured. The cellular components of PRF are leukocytes, platelets, macrophages, granulocytes, neutrophils and erythrocytes. These cells play a fundamental role; in fact, platelets intervened in the first phase of healing in hemostasis with leukocytes, macrophages, granulocytes and neutrophils and participated in the anti-inflammatory phase. Platelets and macrophages release a large number of growth factors within 7–14 days, including platelet-derived growth factor (PDGF), transforming growth factor-β1 (TGF-β1), insulin-like growth factor (IGF) and vascular endothelial growth factor (VEGF) [150,151,155,156,157]. These bioactive molecules are able to further promote cell proliferation and bone remodeling [173,174]. The architecture of the fibrin matrix influences the trapping/release of GFs [156]. The cellular content and the fibrin matrix differentiate between the materials derived from the platelets because these are the elements that are involved in the regeneration and healing of the tissues. Therefore, it is necessary to recommend the use of this method of peripheral blood derivatives PRF associated with biomaterials [157]. When the use of PRF is included, the results indicate that osseointegration and bone regeneration takes place in a short time—only 106 days [147]. Currently, L-PRF as a component of the large PRF family has the potential to biostimulate bone regeneration and accelerate osseointegration [175]. The PRF does not induce new bone formation directly, but what all authors agree on is that PRF significantly improves early vascularization in bone grafts [147,150,151,155,156,157,176]. The PRF through the angiogenesis promoter (VEGF) induces the vascularization of the area and decreases the inflammatory reaction around the graft since it contains anti-inflammatory cytokines (IL-4) [177]. PRF improves soft tissue healing when in contact [178,179,180]. Indeed, some studies have come to the conclusion that PRF promotes and improves soft tissue regeneration more than hard tissue and therefore consider PRF to have slight potential in the GBR technique (guided bone regeneration). Excellent results have been obtained on soft tissues, even from the use of a mixture of ozolipoilein, which, in only 3–5 days, can heal small ulcers caused by radiation therapy cycles. This blend is also antibacterial and antifungal [181]. Instead, PRF significantly contributes to tissue healing through its antibacterial properties, especially against S. aureus and E. Coli. However, this is mostly expressed when a horizontal centrifuge is used to produce PRF [182]. The use of PRF as a membrane has produced effective results in both bone regeneration and soft tissue regeneration. This membrane can be obtained in a non-invasive way and is considered a protective barrier of newly formed tissues. For the patient, this method is very comfortable [183,184]. The PRF can be considered a very valid barrier membrane between the oral cavity and bone, with an easy and predictable preparation protocol and a well-defined structure with impressive mechanical properties, as reported by several authors [147,150,151,155,156,157,158,159,160,185]. A PRF membrane can also be used many hours after preparation as the release of PDGF continues to be high, as long as the membrane is properly prepared in a PRF box and stored under physiological conditions. The release of VEGF and β-TGF significantly increases in the first four hours, but then the amount of these growth factors does not show large differences between blood derivatives because GFs are mainly derived from leukocytes [186]. A new generation of blood products called CGF from Silfradent is produced without anticoagulants. The MEDIFUGE MF200 centrifuge that produces CGF is from Silfradent, has a special multi-speed centrifugation program, creates a gradual sinusoid (RPM) at various speeds and offers a large, high-density fibrin matrix rich in GFs [187]. This CGF is composed of three layers, a dense fibrin network and blood cells trapped within it, especially in the most outer part [188]. The first layer is called PPP and is a network of plasma proteins in which cells without a nucleus are found. These cells seem to have the typical appearance of erythrocytes. The second layer of CGF is much denser than the first and third layers. It is a fibrin network, in which cellular elements are trapped and collagen fibers are found where other corpuscular elements are captured. The CGF layer consists of three fragments: the upper white part, the lower red part and the buffycoat at the interface between the white and red part [187]. The third layer of RBC red blood cells appears to have a wider network of collagen fibers than the second layer, and coreless corpuscular elements, such as platelets, are found entangled in this network [188]. The CGF fibrin matrix is denser than PRF and has GFs present in it, such as TGF-β1 and VEGF, which are important in cell proliferation. The presence of these growth factors was also identified in the RBC layer. The CD34+ cells are trapped in the CGF matrix with a considerable number of them and have been found in both levels (CGF-RBC). The CGF appears to be more promising in tissue regeneration, as it promotes osteogenic cell differentiation and proliferation. Therefore, with the CGF, the activity of ALP [162,189] significantly increases. CGF also appears to be very promising in neural tissue regeneration. The controlled release of growth factors and cytokines from CGF influences cell differentiation and intervenes in the growth of neurons [190]. In conclusion, as a result, we have the PRP with a lower concentration of GFs compared to A-PRF or CGF. Advanced Platelet Fibrin (A-PRF) and Concentrated Growth Factors (CGFs) contain TGF-β1, VEGF, PDGF-BB, IL-1β and IL-6. Masuki et al. said that the main source of PRF growth factors is in the exudate and has a very low percentage of GFs found in the fibrin network [191]. However, Ehrenfestet al. suggest that exudate is not the most important part in platelet concentrates [159]. Kawaseet al. reported that even for PRP, which is in a liquid state, fibrin gel and thrombocyte aggregates, many more biological mechanisms will emerge [192,193]. This is also accepted from other studies [150,151,155,156,157,158]. An effective method used for bone reconstruction is the penetration of CGF into the Xenograft blocks with the method that uses the Round Up device from Silfradent [161,187]. The same method is also used with β-TCP, which shows that this combination significantly increases the release of certain growth factors, such as BMP-2 and BMP-7. Scientific evidence shows that calcium ions that are released by the dissolution of β-TCP influence the activation of platelets [150,151,155,156,157,158,159,160]. The Ca_2_+ ions stimulate the differentiation of osteoblasts and increase the stability of BMP-2. The same study reveals that CGF used alone instead of a combination increases the release of IGF-1 [194]. The release of growth factors is high in the first few days then starts to slow down. With the aim of overcoming one of the main limitations of PRF and its degradation, an in vitro study was conducted on Alb-PRF, an injectable biomaterial, produced by PRF in liquid form and PPP heated to 75 degrees Celsius in 10 min with a device called APAG [187,195,196,197]. Since PPP is composed of 60% albumin, its denaturation creates an organized and dense protein structure without cells. This combination with APAG has been shown in vitro to preserve the volume and prolong the reabsorption times, leading to that the conclusion that the release of growth factors can continue much longer than in the traditional PRF [187,196,197]. The Alb-PRF preserves its volume even after 21 days [198]; however, the Alb-PRF membrane loses the elasticity of the PRF membrane and becomes very fragile. To overcome this deficiency, the use of high-power laser pulses is suggested [199]. Laser irradiation is performed on a surface, leaving the inner part of the membrane intact. This method seems to stimulate and increase stability in the body and allows for better suturing/fixing of the membrane, thus optimizing the Alb-PRF [199]. Even with the albumin denatured with CGF, a solid and dense membrane of cells with nuclei is obtained. This Alb-CGF membrane is capable of releasing growth factors (VEGF, PDGF, FGF2) for seven days [195]. A comparative study was conducted on the preservation of the alveoli after extraction (socket preservation) using only Alb-CGF and allograft covered with albumin. This study demonstrates that radiographically and histologically, there was no significant difference between the two groups tested; thus, Alb-CGF is considered a promising biomaterial in tissue regeneration. In fact, the authors conclude that Alb-CGF gives the same results as a bone graft with allografts and albumin (sticky bone). This material is believed to be capable of immediate and prolonged release of growth factors [200]. Other studies have reported the effectiveness of CGF and PRF in osteogenesis in patients with systemic diseases. Systematic diseases are those that must be taken into consideration in particular for bone regeneration because, in these diseases, there is the need for a personalized protocol according to the underlying pathologies and the planning of the work to be carried out. The use of bisphosphonates in patients with osteoporosis reduces the production of BMP-2, while CGF or treatment with resveratrol combined with CGF instead promotes the production of BMP-2, and they have a positive role on osteoblasts in patients treated with bisphosphonates [150,151,155,156,157,158,159,160,201]. Bisphosphonates induce bone growth, but they alter bone turnover, decrease osteoclasts and significantly reduce vascularization in adult patients [202]. Moreover, in children and young patients, they are protected from this phenomenon [203]. Due to the apoptosis of endothelial cells, bisphosphonates decrease the number of endothelial progenitor cells, so both vascularization and neo-vascularization are reduced [204]. On the other hand, the use of CGF in combination with sodium orthosilicate has been shown in vitro to stimulate cell growth, proliferation and metabolic activity in several human cell lines, including fibroblasts, endothelial cells and osteoblasts [205], and that the use of growth factors, such as epidermal growth factor (EGF), may partially neutralize the effects of bisphosphonates on human oral keratinocytes and human umbilical cord endothelial cells via the EGFR/Akt/PI3K signaling pathway [206]. Diabetes is another very widespread systemic disease. Patients with diabetes have a noticeable reduction in the potential for healing and vascularization of the affected tissues. Durmuşlar et al. concluded that the use of PRF alone did not produce significant good results; only in combination with autologous bone does PRF induce bone formation in diabetic rats and significantly increase bone volume compared to the group where only autologous bone was used [207,208,209,210,211,212,213,214,215,216,217,218,219,220].

## 6. Biostimulation and Laser–Graft Interactions

In daily practice, the use of Lasers, Light Amplification by Stimulated Emission of Radiation (Table 2), is increasingly being applied. Its use leads to an operating field cleaner from blood and atraumatic work. Additionally, it significantly reduces pain and postoperative edema [221,222,223,224,225]. The use of lasers offers better aesthetic effects compared to other techniques since it does not leave an obvious scar, giving the part of the body where the intervention is performed an excellent restitutio ad integrum [221,222,223,224]. Photodynamics includes a photochemical and non-thermal biological interaction. Photobiomodulation (PBM), also known as low-level laser therapy (LLLT) [226], is a safe and non-invasive clinical method that, in most cases, uses red 600–700 nm and infrared 770–1200 nm light, with the aim of stimulating healing and reducing inflammation and pain [227]. The mechanism of action of the LLLT is based on the interaction between chromophores cells (especially the enzyme of the mitochondrial respiratory chain; cytochrome C oxidase) and laser light. The absorption of light by these chromosensors generates more energy (ATP) and, in addition, causes the modulation of the calcium level and an increase in the production of nitric oxide [227]. The LLLT has been recommended by the Multinational Association of Supportive Care in Cancer/International Society of OralOncology for the treatment of oral mucositis in adult cancer patients, but LLLT has been shown to reduce severe mucositis even in young cancer patients [228]. The use of low-intensity lasers has also given excellent results in many oral diseases [229]. In recurrent aphtosis and herpes simplex, it reduces pain and provides complete wound healing, and when combined with antibiotics, improves the condition of BRONJ/MRONJ patients by reducing clinical symptoms in hyposalivation or xerostomia. Therefore, LLLT is very effective in cell repair and increases secretion by stimulating the salivary glands. LLLT also reduces the symptoms of trigeminal neuralgia [230,231,232,233,234,235]. The use of LLLT has also spread widely in the field of periodontology and peri-implantology [236,237], but the effects that lead to the use of long-term laser on scaling and root planing (SRP), bacterial-subgingival reduction and sulcular debridement (laser curettage) appear to have no significant advantages over other methods [238]. However, the use of the laser gives excellent results on the surfaces of implants that have been attacked by peri-implantitis and have part of the fixture exposed in the oral cavity. The use of the laser decreases the microroughness and porosity of the implants, thus giving a more glossy surface, which prevents the adhesion of bacteria on smooth surfaces [225]. In their study, Pereira et al. reported that the use of the LLLT did not lead to any change in bone regeneration compared to the control group, but there was a noticeable difference in the interface between the implant and the bone, thus allowing for better osseointegration [239]. Grassi et al. reported that the use of LLLT promotes proliferation and differentiation by significantly increasing cell adhesion on the implant surface [216]. The ability to promote osseointegration and stimulate bone formation around the implant has also been confirmed by other authors in their research [240,241]. Other studies have also been developed to test the effect on mechanical strength that occurs during the use of the LLLT laser [242,243]. Maluf et al. tested two groups of mice: one group received laser therapy and the other did not. This study concluded that the group of mice that received laser irradiation had greater difficulties detaching the implant from the bone than the group that was not irradiated. A torque machine was used to measure the torque needed to detach the implant from the bone. The result showed that the group treated with laser had a resistance of 50% more than the other group without laser irradiation [242]. Other studies have used the LLLT laser to enhance osteoinductive properties in biomaterials that only possess osteoconduction [212,213,244,245,246]. For this purpose, the biphasic HA/TCP and bovine bone were tested as a scaffold. The groups to be tested were irradiated every 48 h for 13 days of 40 s per sector and, after 60 days, with a second intervention the endosseous implants were inserted. The results showed significant differences between the control group and the group tested 15 and 45 days after endosseous implantology. In this experiment, increased exposure of BMP-2 and osteocalcin was observed [213]. The authors concluded that the LLLT promotes osseointegration with a high expression of BMP2 and osteocalcin related to osteoblastic activity and that the force required to remove the implants could be compared with that of the implants inserted in autologous bone [213]. Another study that tested the use of LLLT on bovine bone (DBB) and biphasic HA/TCP led to the same conclusion. After 90 days in the irradiated group, there was bone augmentation, with an increase in the expression of BMP2, Osteocalcin (OCN), alkaline phosphates (ALP) and genes [212]. The LLLT laser is described as a promising therapy for stimulating osteoblastic activity and differentiation. Furthermore, it has been observed that LLLT improves the osteoconductive properties of these materials while maintaining the volume of the bone graft [212]. Other studies have used photobiomodulation to repair bone defects with bovine bone grafts. The irradiation began on the day of the surgery, and then seven other irradiations were performed every 48 h [244,245,246]. The use of LLLT in these studies led to a significant increase in the deposition of collagen in the first healing phase, as well as greater osteoblastic activity, rapid bone remediation with neo-formation of the Haversian system and regeneration of the cortex [244,245,246]. Another study, which lasted 12 months, used enamel matrix protein-derived (EMD) combined with laser LLLT in an intra-osseous defect. The radiation was performed after the surgery with a 4 J/cm^2^ diode laser for five minutes on each side of the defect in five days. The result showed that the use of LLLT improved the effect of EMD in the intra-bone defect [247]. The light-emitting diode (LED) is another promising alternative for tissue biostimulation. Unlike the LLLT, which emits coherent light, the LED has non-coherent light. The LED has a lower cost, is more manageable and does not carry the same risk to the eyes. The LED appears to have the same effects as the red light laser on the stimulation for the growth of pre-osteoblasts [217], promotes osteogenic differentiation and inhibits cell proliferation [248]. The application of the LED together with photosensitive substances has also led to excellent results in the osseointegration of implants inserted into bone diffusers and filled with biomaterials. After some time, the bone formed around the implants can be compared to autologous bone [249]. However, the use of LEDs is limited; for a small area when a high power density is required, it almost always requires the use of lasers [250]. Mergoni et al. conducted an in vitro 915 nm laser study on human osteoblasts. The treatment did not provide improved effects on cell proliferation and differentiation compared to the non-irradiated group but induced the formation of bone nodules by a considerable amount [219]. Instead, Jawad et al. concluded that decreasing the laser power increases osteoblastic cell differentiation and cell proliferation increases as the laser power increases. Thus, the cell proliferation of osteoblasts using a 940 n diode laser increased more when the irradiation power was 300 mW compared to 100 mW, while the expression of ALP and osteocalcin was, respectively, higher with the radiation at 100 mW and 200 mW compared to 300 Mw [220]. Barbosa et al., In their research, made a comparison between red light lasers and infrared lasers. They claim that the use of the infrared wavelength laser gives better results compared to the red wavelength laser, thus concluding that the bone healing process depends on the time and wavelength [251]. This result also agrees with the conclusions of Queiroga et al. [218] and Renno et al., which also confirm the fact that different cell lines respond differently to specific combinations of wavelengths and doses [252]. Tani et al. tested biomodulation with three different wavelengths: red light (635 nm), infrared light (808 nm) and LED (405 nm). The aim was to evaluate the differences in proliferation, adhesion and differentiation of pre-osteoblastic and human mesenchymal stomal (hMSC) cells [253]. Bone nodules (Ca_2_ deposition) were observed differently in both laser wavelengths from the radiation with LEDs. On human mesenchymal stromal cells, the 635 nm wavelength is capable of promoting osteogenic proliferation, adhesion and differentiation, while on osteoblasts, this wavelength does not have a significant effect on cell differentiation by questioning red light (the wavelengths) or the parameters used for radiation (0.5–1–2 J/cm^2^). Moreover, this study proposes the diode laser with a wavelength of 635 nm as an effective option to promote and stimulate bone regeneration [253]. On the other hand, Ghidini et al. concluded that red waves (645 nm) could penetrate to considerable depths [254]. All these data show that there is still a great deal of confusion on the working protocols for biomodulation. Many authors suggest that the use of LLLT fundamentally improves and accelerates the healing processes of newly formed tissues [208,209,210,211,212,213,214,218,219,220]. These conclusions have already necessitated a recommended and studied protocol of a guideline with irradiations and the type of laser prescribed by the World Association for Laser Therapy (WALT) on biomodulation, as its positive effects certainly cannot be denied [255].

## 7. Microbiota in Tissue Repair Processes

Other factors come into play in bone and soft tissue metabolism. The crucial role of the intestinal microbiota in the regeneration process of our organisms is the subject of great study and debate. Several authors agree in asserting that the intestine as a whole is the place where basically any dysfunction begins, even before a symptom or pathological event is diagnosed or even evolved. The balance/imbalance between the constituent parts of the gut is known as symbiosis/dysbiosis, which accurately reflects systemic health. A healthy intestine is made up of the balanced presence of four Phila bacteria: *Bacteroideti*, *Firmicutes*, *Actinobacteria*, *Proteobacteria* and *Verrucomicrobia*. *Ibacteroideti*, and firmicutes represent the majority of the microbiota [256,257]. Over the past two decades, one of the most significant scientific discoveries has been the confirmation of the Neuro-Endocrine-Immune nature of the intestine, which as a single apparatus tends to perform an activity believed to be exclusive to the central nervous system (CNS), so much so that nowadays, it refers to the gut–brain axis. In fact, the two systems share anatomically and physiologically important multi-functionalities and have neurochemical, endocrine and immune significance that makes the intestine an autonomous system called the enteric nervous system (ENS), which is responsible for all gastrointestinal activities in full coordination with the CNS [258]. A further aspect that has been highlighted is the inter-communicative capacity of the two systems through a very complex network outlining a common clinical-pathological picture. In fact, many important pathologies tend to afflict the two systems in a one-to-one and constant way [259,260]. Within the intestine, the microbiome functions as a guarantor of the homeostasis of all the other systems of the organism, capable of producing 50 to 100 mmol/L per day of short-chain fatty acids (SCFA), such as acetic acid, propionic acid and butyric acid, which are the sources of energy for both the proper functioning of the complex cellular structure that makes up the intestinal epithelium and for the constant maintenance of the skeletal system [261,262,263,264]. The importance of SCFA among acetic acid, for example, is essential against infections, in regulating blood pressure and against the deposition of sclerotic plaque in the arterial walls. Butyric acid, for its part, acts as an inhibitor of inflammatory responses thanks to its immunomodulating properties, while propionic acid has been found vital in the prevention of obesity and diabetes 2 [261,262,263,264]. Furthermore, the intestinal system performs a “prompt delivery” function of vitamins, such as folic acid and the vitamin B_2–12_ complex, and at the same time, it allows the synthesis of vitamin K, which is involved in many physiological, metabolic and immunological activities and plays a fundamental role in the prohormone/vitamin D absorption mechanism, which is important for the homeostasis of the skeletal system and the formation of osteocytes, osteoblasts and osteoclasts [263,265]. The way in which this interaction takes place and the mechanism in which they carry out these processes reveals something that is revolutionizing the conventional thinking of contemporary medicine, for which we refer more and more often to the term epigenetics [266].

Epigenetic events are highly dynamic and change in response to the availability, quality and quantity of environmental insults both external and internal to the oral cavity. In this perspective, there is a tendency to have a substantially different perspective in which bacteria, viruses and fungi, which are also harmful and dangerous, are still indispensable for life. The results of the experiments conducted on germ-free (GF) mice confirmed that the shortage of gut microbiota was to be indicated as a functional cause of the severe deficiency of the immune system. GF animals were shown to have low levels of natural killer cells (NK), dendritic cells (DC), α/β + and γ/δ + T cell populations, which play an important role in defense and pathogenesis during inflammation and infection, particularly against some types of malignant neoplasms. Furthermore, the above experiments have unequivocally demonstrated that GF animals were particularly susceptible to frequent infections substantially due to the decline of angiogenin-4 (Ang4), a powerful antimicrobial molecule belonging to the class of microbiocidal proteins in Paneth cells [267,268].

### Dysbiosis and Metabolic Disorders Related to the Bone Tissue Degenerative Processes

What is the connection between dysbiosis and the bone system? The conclusions revealed a different type of bacteria. In fact, patients suffering from osteopenia revealed an increase in the number of *Firmicutes* phyla with a reduced number of *Bacteroidetes* compared to patients in the control group; the genera *Lachnoclostridium* and *Klebsiella*, *Gemmatimonadetes Chloroflexi* and *Synergistetes* were detected to a high extent in both patients with osteoporosis and in those with osteopenia but were absent or very low in the control group; the control group showed a prevalence of *Bacteroides*, *Faecalibacterium and Prevotella*. Interestingly, *Prevotella* was in a constant presence in the osteoporosis group but very low in the osteopenia group [269,270,271,272].

In patients suffering from Crohn’s disease and ulcerative colitis, recent studies have confirmed the coexisting presence of osteoporosis, and the cause can be found in a chronic inflammatory state. One of the mechanisms involved seems to be related to the immune-mediated bone metabolism involving the RANKL axis (an activator of the NF receptor kappa B ligand NFκB-RANK), the osteoprotegerin (OPG) and the basic activation of the immune-receptor of the tyrosine (ITAM), all members belonging to the TNF super-family and all factors sharing the same androgen signaling pathway [269,270,271]. The chronic overexpression of pro-inflammatory interleukins determines inhibitory activity towards hormones, such as estrogen (E2), testosterone and progesterone, which play a key role in the formation, production and stabilization of such bone tissue. The decreased intake of these hormones triggers a vicious cycle of worsening the equilibrium state of the immune response and increasing the inflammatory quotient to almost block physiological bone turnover completely. This scenario is often found in women going through menopause and in men over 60 who present a physio-pathological picture that recalls the typical picture of many autoimmune syndromes and of different neoplastic forms [273,274,275]. The increase in phlogosis, the expression of inflammatory interleukins, such as interleukin 1α and 1β, IL-6, IL-11 and IL-17, also contributes to the decrease in the pH, which becomes peremptorily acidic and also contributes to slowing down the differentiation of mesenchymal cells from osteoblasts. In fact, it is well known that this process from MSCs to osteoblasts can only occur in an alkaline physiological environment. An interesting fact is the relationship between OPG and MSCs; both intervene in the regenerative phase by containing osteoclastogenesis precisely through their blocking action on RANK via RANKL. This ultimately explains how in a state of imbalance, determined both by a chronic inflammatory state and by the lack of male and female steroids, such as estrogen, testosterone and progesterone, the regenerative process of the bone tends to undergo a major arrest at the expense of remodeling activity on osteoclasts [276,277,278,279]. It is now known that not only intestinal dysbiosis but also oral bacteria, particularly periodontal bacteria, influence osteoporosis and bone loss. The oral microbiota plays an important role in many systematic conditions and vice versa [258,272,280,281,282,283,284]. Except for oral hygiene, which helps maintain the physiological state of saliva, a balanced diet and supplements of non-pathogenic microorganisms must be used to keep the oral microbiota in the normal range [285]. These non-pathogenic microorganisms are named probiota, and by interacting with the intestinal microbiota, have given good results in various conditions and pathologies [259,265,286,287,288,289,290].

## 8. Stem Cells Therapies for Regenerative and Translational Medicine

The direct use of stem cells requires a studied, planned approach and, above all, one that takes into account the general condition of the patient. For doctors, orthopedists and dentists, the use of pluripotent and multipotent stem cells, such as MSCs, constitute a superior quality method that exponentially improves the body’s regenerative capacity and ability. In fact, stem cells (SCs) are generally able not only to differentiate into new tissue but also coordinate, modulate and manage the entire chain of events of both repair and regenerative responses—just think of their role on M1 pro-inflammatory macrophages [291,292,293,294,295,296]. The M1s under pressure from MSCs trigger the recruitment of interleukins and pro-inflammatory cytokines involved in the first phase of the repair process, such as IL-1β, IL-2, IL-6, IL-17, TNFα and IFNγ, while M2 initiate recruitment in the second phase of anti-inflammatory interleukins, such as IL-10, IL-4 and IL-13, prostaglandin E2, oncostatin and bone morphogenetic protein 2 (BMP-2). This mechanism is of crucial importance, especially in the presence of the inhibitory effect of some autoimmune disorders on M2, in which the bone tissues are compromised and the regenerative process is completely blocked [297,298,299]. Preparing the laboratory procedures for bone regenerative therapy for SCs requires much more than a simple study centrifuge. The SCs are isolated and cultured in a sophisticated sterile cell culture laboratory by cell biologists, usually for a period ranging from a few days to two weeks. SCs can be obtained from different sources and can be both autologous and heterologous (from a donor). The main sources are peripheral blood (PB-SCs), bone marrow (BM-SCs), adipose tissue (AT-SCs), dental pulp (DP-SCs) or placenta (Pl-SCs), amniotic fluid (FA-MSCs) and umbilical cords (UCB-SCs). The efficacy of SCs from BM, AT, DP, UCB, Pl, FA or PB alone or in combination with bio-implants is a vast and expanding field of research and study. The information and data obtained are all in agreement in confirming the immuno-modulating and regenerative properties of SCs with evident improvements in anti-inflammatory, osteogenic and angiogenic activity. Part of this effect is surely due to the intrinsic ability of stem cells to secrete anti-inflammatory and angiogenic cytokines and growth factors, such as IL-10 and TGF-beta [297,298,299,300]. Peripheral blood has shown some advantages over other solutions for everything related to the use of PB-SCs for clinical purposes in vivo. From the peripheral blood, the cells are easy to harvest with a minimally invasive procedure in numbers and quantities readily available and with a low risk for any immunological reaction or rejection. Furthermore, PB-SCs do not require prolonged periods of in vitro culture, retain their multipotent capacity and remain substantially able to differentiate into different cellular phenotypes [297,298,299]. These cells were photographed with an electron microscope and then morphologically evaluated, and they were then tested and carefully characterized with the RT PCR method to identify the expression of genes such as Oct4, Sox2, OCN, Nestin, Nanog and DMP, all highly specific genes of pluripotent and multipotent SCs [297,298,299]; confirmations in this sense were then obtained with fluorescence analysis for the expression of genes such as TRA-1 and CD-44; flow-cytometric analysis then confirmed the trend in which both adherent and non-adherent mononuclear cells showed positivity with a panel of multipotency and pluripotency markers, such as CD44, CD73, CD90, CD133, CD34, CD45, CD14, Nestinand SSEA-3 and TRA-1, of NSCs. In addition, it was possible to perform a hormone quantification analysis from both female and male donors, and the results showed the presence of 14 hormones in the extracellular matrix component, the major ones being testosterone, estradiol, progesterone and cortisol. The isolated adherent and suspended mononuclear cells were able to maintain their plastic properties during in vitro culture while maintaining their ability to proliferate and differentiate when exposed to the appropriate culture medium [299]. The regenerative, restorative capacity of these SCs has been proven by multiple studies, especially in the area of the osteo-skeletal system. Recently, a study explored a new solution for osteo-regeneration at the level of the mandibular and maxillary bones with a resolution composed of the combination of β-TCP granules, autologous peripheral blood fibrin (hPB) and autologous peripheral blood stem cells [82]. The isolated PB-SCs were directly transferred and inserted into the previously constructed fibrin and β-TCP scaffolds. The β-TCP granules with diameters of 1 mm and 1–2.5 mm were embedded in a fibrin gel matrix and subsequently cultured with serum-free medium (SFM) for a period of 7–10 days. The compound was named compact bio-bone (CBB) [82]. In vivo CBB induced both horizontal (Table 1) and vertical (Table 2) growth, but the most important aspect was the quality and compactness of the new bone tissue, which revealed a formation of solid and compact tissue very similar to the structure of the original endogenous bone. The histological analyses carried out then, in fact, confirmed the presence and formation of new lamellar bone in excellent condition with the presence of osteocytic islets in lacunae and clusters of active osteoblasts in the production phase of a new matrix [82]. Understanding the tissue microenvironment in which you intend to perform an implant, a graft or simply inserting supports to facilitate bone fusion has certainly conditioned a new way of doing Regenerative Medicine, supported by in-depth studies on both the choice of elements and on the selection of innovative biomaterials to be used in the operating room. However, the use and continuous experimentation with new materials and new solutions had to deal not only with the vision of the mechanical properties of the tissue to be recovered or regenerated, be it bone, tendon or skin, but that of being fully compatible and similar with the part in which it is inserted keeping the surrounding structures intact [17,60,301]. An attempt was therefore made to broaden the overall vision by trying to identify the metabolic, genetic and decline conditions of the recipient because the failure of a specific implant or regeneration procedure also includes a broader and more careful analysis of the conditions that led to the process of degradation or rupture of a specific structure, such as bone [60,104,302]. In fact, a careful phase of tests and clinical evaluations was necessary, which must have the individual as its center in its complexity, and this epistemological attitude will therefore not only be focused on the quality of new and increasingly compatible bio-materials and inductive fabrics but will have to ignite a new perspective, on the importance of the physiological-molecular processes involved in the disease state intended as a progressive decline of the vital activities of the organism. Much remains to be learned about the close relationship between the microenvironment of bone tissue, the process of degenerative states and the patient’s external environment in order to evaluate a compatible and individualized treatment [14]. 

## 9. Bone Substitutes and Teeth Graft

Few studies evaluated the use of the autologous tooth in bone regeneration, where there is little evidence on the use of this biomaterial. Based on the properties that this material possesses and what it can offer in bone regeneration, there are great expectations in this regard (Table 3 and Table 4), but further studies are still needed [98,303]. 

Calcium phosphate creates new bone faster than bovine bone [314,315], but they are also reabsorbed faster. The bovine bone preserves its volume for a long time by keeping the apex of the implant covered. The Calcium triphosphate (TCP) loses its volume, and the apex of the implant is exposed [307,310,311]. Yoko et al. believe that this loss of volume does not lead to any contraindication because this resorption is related to the length of the implant inside the sinus, and after three years, it is osteointegrated [311,312]. Oliveira et al., in a comparative study between TCP and inorganic bovine bone, stated that the implants have better primary stability in patients where only bovine bone was used [314]. For a different result, these materials can also be used combined together [316] or with blood derivatives PRF [160]; however, PRF improves the primary healing of soft tissues but does not give long-term benefits on hard tissues [305,317]. The use of the BMP morphogenic protein has given very promising results [318]. Although BMPs have a strong osteoinductive potential, used alone, they are very soluble and lose their properties. The combination with biomaterials serves as a carrier for BMPs. The BMP is found naturally in the dental matrix [98,111].

In the case of tooth extraction, a loss of approximately 30–60% in horizontal width and 10–20% in vertical length of the bone is expected after six months compared to the volume before tooth extraction [328]. Very few studies have been conducted on calcium triphosphate (TCP) in humans. This material resorbs completely, and the resorption time is rapid and fails to retain volume. It is often suggested to use it in the form of composites with a collagen matrix [75,329,330] or with calcium sulphate, which has given good results [327,331]. Compared to TCP, bovine bone preserves the volume longer because it has more graft residues, but it has less vital bone [331]. A rare case of a human autopsy was carried out five and a half years after the operation. The guided bone regeneration technique, GBR, was applied to the patient using biphasic calcium phosphate and autologous bone in contact with the implant and then using Bio-Oss bovine bone. The result of the autopsy shows that Bio-Oss had preserved the bone volume, but in the meantime, autologous bone with biphasic calcium phosphate had lost bone volume. The vestibular bone was concave, and recession of the mucosa was also seen [332]. Carmagnola et al. observed that the quality and quantity of bone added with the bovine graft was sufficient for the preservation of the alveolus and that the implantology and regenerative procedure was performed correctly [333]. However, the residues of the bovine graft remain for a long time without being reabsorbed, and consequently, there remains the suspicion that the implant at the time of contact with the bovine bone residues has osseointegration problems [334]. The dental matrix has a porous microstructure that promotes cell adhesion, blood circulation and slow resorption that guarantees correct osteoconduction, thus ensuring that the bone volume is preserved for longer [303]. Joshiet al. states that the autogenous tooth material has given good results compared to TCP while maintaining the length and width of the alveolar ridge after extractions [335]. In another analysis, the tooth material was found to be in close contact with the implant surface [336], making the dental matrix a promising biomaterial in preserving the alveolar ridge [98]. Kabir et al. removed the enamel and pulp from the tooth, making holes in it for use in critical defects. After four months, the new bone formed around the dentin graph and inside the pores was identified. This demineralized dentin microstructure showed osteoconductive and osteoinductive properties [337]. Schwarz et al. used autogenous teeth in an animal study for lateral alveolar ridge growth. The teeth were cleaned of cement, and the crown was cut. After endodontic treatment, the canal was filled with Ca(OH)_2_ in one group and the tooth was not treated endodontically in the other. Root dentin was used en bloc to fill the bone defect. To compare the results, they used the bone block obtained in the retromolar region. The authors concluded that the presence of dental pulp made no difference in bone regeneration compared to the endodontically treated group. The grafts are gradually reabsorbed to be replaced by homogenous bone intertwined with parallel fibers, and the dentinal block in this case has given good results for the lateral augmentation of alveolar bone followed by the subsequent positioning of the implants [338]. In the continuation of this experiment, Becker et al. reported that the dental block as a bone graft has a greater exposure and a higher failure rate than autologous bone [339]. Pohl et al. compared the dental block with the dentin particles for the lateral growth of the alveolar ridge. The authors state that the tooth material can be considered a good alternative in bone regeneration, but the dentinal block compared with the dentinal particles had distinct, clear bone margins and no macroscopic signs of bone remodeling, indicating very slow resorption [119]. Due to biocompatibility and bioactivity biomaterials of synthetic origin are widely used, but their study must be expanded in their compound form or with the combination of biomaterials or substances derived from macromolecules. Jack and Lemon state the need for these biologically derived substances [340]. For defects larger than two centimeters (>2 cm), it is recommended the use of bioactive molecules, such as growth factors, peptides of the extracellular matrix (EMC), and small molecules, such as Parathyroidhormone, Nel-like molecule-1 and LIM mineralization protein-1 [341]. Research has been developed for a long time to finalize and improve the mechanical properties and osteoinductivity of alloplasts. The combination of HA conf MWCNT 0.5% wt appears to be an appropriate combination as it improves mechanical strength and hardness; however, it remains a bio-scaffold with osteoconductive properties even though it has been claimed that this combination gives a scaffold with magnetic properties that will help to bring the growth factors into the implant site [77]. There are authors who give importance to osteoconductivity [342,343], but others think that poor osteoinductivity has often led to lower expectations in bone regeneration [175,344]. However, the third generation of bone grafting materials continues to develop more and more on the search for scaffolds capable of inducing cellular responses (Table 5 and Table 6) [60,345]. The use of human growth factors and stem cells together with biomaterials is believed to improve the osteoinductive properties of the scaffold [346]. Harwood et al. support the idea that the lack of growth factor secretion leads to delayed bone healing or even non-union with the host site [347]. Compact Bio Bone Cell increased osteoinductivity using stem cells [82].

However, these bio-scaffolds have a high preparation cost. Synthetic HA and TCP need further studies to improve their mechanical and osteoinductive properties. The new alloplast osteoinductive Osopia seems to complete the three osteoinduction formation criteria, generating ectopic bone in the end. However, it is too early to discuss this biomaterial as further studies are needed to prove and determine its properties [80]. Calcifications of the bovine and dental matrix are considered, from the physiological point of view, as the most acceptable processes of HA production [348]. Bovine bone has a high resistance in the scaffolding, and its porosity allows cell circulation and the formation of angiogenesis. The reabsorption of this material remains very slow [143]. Xenografts do not have the ability to recruit or differentiate mesenchymal cells, so this biomaterial cannot produce ectopic bone. The increase in growth factors in this material has given positive results because xenografts are devoid of cells and proteins. The application of GFs has been extensively studied, and some of these are involved in the bone regeneration process, such as the BMP bone morphogenic protein [349]. The use of BMP-2 with other reconstructive materials recruits MSCs to improve differentiation in mature osteoblasts. When the BMP is used correctly, the regeneration of complex bone defects is possible, and BMP also reduces healing times [115], but these growth factors that participate in particular in osteogenic differentiation and cell interaction need to be explored in further studies because they are of great importance in bone regeneration [350]. Bracey et al. stated negative results using bovine bone as restorative material [165]. Out of ten cases considered, four of them failed in a period of 24 months. The etiology of this biomaterial is considered as a strong cause of the failures, and the immune response of the host body (anti-Gal antibody) led to the rejection of the implant because it is not possible to eliminate 100% alpha-Galepitop antigens. This review suggests using this biomaterial less and less [164]. The autologous graft remains the gold standard in bone regeneration [351], but due to the morbidity it causes and the limitation of the amount that we can obtain, studies continue to look for a replacement material [352,353]. Today, more and more attention is paid to autologous dentin grafts, which eliminate the risk of antigenicity [96]. An autologous tooth can be used by the same patient even after a long time from its avulsion, and the dental element can be preserved without additional liquids. Schmidt-Schultz and Schultz confirm that the tooth retains the inorganic matrix like the organic one even after hundreds of years [354]. If the dental matrix is processed in the right way, it has osteoinductive properties determined by the morphogenetic proteins BMPs, which are contained in physiological quantities in the tooth. The tooth crushed with Tooth Transformer (TT) seems to preserve these osteoinductive properties with a partial demineralization of the dental matrix without damaging its structure, offering a graft in 400–800 mm particles that are easy to handle during surgery [98,123,125,128,130,131,132,134]. The demineralized dental matrix has a faster resorption time than mineralized dentin as biodegradation is almost impossible for large particles with high crystalline. Small-sized HA crystals show the best osteoconduction effects [98,129,130,132]. In terms of comparing biomaterials, the healing time that each material takes is important, as it shows a great effect of new bone formation. Autogenic bone appears to be more effective in the first six months, but for a longer healing time, different materials achieve similar histomorphometric results between the various biomaterials. This means that when a surgeon has no time limit and can therefore wait more than six months for loading, can choose several alternatives, which give similar results to the autogenous bone, but the use of GFs and MSC in combination with other biomaterials increase and improve the healing rate [355].

## 10. Hyaluronic Acid and Bone Regeneration

Hyaluronic acid (Ha) is a natural extracellular matrix element that could be a promising component for the regenerative procedures for hard and soft tissues [356,357]. In literature, it was demonstrated that the Ha is able to improve cell adhesion, proliferation, and differentiation [358]. The treatment of the maxillary bone defects often requires a grafting approach with scaffold materials in order to restore the anatomy and the function of the damaged soft and hard tissues. In addition to the scaffold biocompatibility, the mechano-physical behavior, biodegradability and bioresorption represent a key factor for successful osteointegration [359]. The hyaluronic acid-based scaffold could play a broad role in improving the scaffold’s biological behavior and other implant materials [359]. Many different combinations of Ha-based biomaterials have been studied, such as Ha–bioglasses, Ha–metals, Ha–inorganic (calcium phosphate, Ha–hydroxyapatite) and organic grafts (polycaprolactone and poly-L-lactic acid) [360]. The principal advantages connected to the hyaluronic acid compounds are increased homogeneous and stable morphology, enhanced mineralization processes and material degradation [358]. Moreover, the hyaluronic acid-based biomaterials are available in the form of colloids, an injectable polymeric scaffold. In colloid form, the Ha crosslinking gradation could affect the mechanical behavior, the architectural characteristics and the material’s stability [361,362,363]. The colloids form could be used as drug carriers by incorporating osteogenic molecules, such as TGF-β, bone morphogenetic proteins and angiogenetic factors, such as VEGF, FGF, PDGF and chemotactic cytokines [364].

## 11. Conclusions

Numerous medical requirements in bone regeneration have made it possible for a wide range of materials. Studies continue in order to offer a product that meets the clinical needs of bone regeneration. To be used as bone substitutes, biomaterials must maintain the volume for a sufficient time until they are completely replaced by bone tissue and resist over time. None of the materials fully meet the requirements for any bone defect. This must be taken into consideration, and depending on the procedure and surgical needs of the bone defects, the materials must be chosen. The choice will be made based on the surgical problem to be treated, the clinical condition of the patient who may have comorbidities or hormonal-metabolic problems and also the needs related to the necessary regeneration time, the size of the defect, the opportunities that each country offers through bone banks and the economic and religious requirements of patients. An increasing amount of literature is focused on the recent applications of non-transfusional hemocomponents to enhance bone repair capability and the local response to tissue repair. These novel techniques could reduce the healing period of the alveolar bone defects. In particular, the use of specific growth factors and BMP in combination with different grafts could represent an advantageous approach to improve the new bone formation for a wide range of defects. The advantages deriving from the use of LLLT and lasers could produce different results depending on the frequency used and the type of regenerative intervention to be performed.

## Figures and Tables

**Table 1:** Summary of the electronic databases Boolean search strategy (PubMed/Medline, PubMed/Central, Web of Science; Google scholar). No limitation about the publication years has been considered.

Databases Search strategy	TITLE-ABS-KEY “((Bone grafts OR Bone substitutes); (Bone regeneration AND Biomaterials); (Bone tissue engineering AND Scaffold); (Bioceramics AND Tricalcium phosphate OR Hydroxyapatite); (Dentine graft OR Tooth graft); (Xenografts OR Bovine bone); (Laser therapy OR Low-level laser therapy OR LLLT); (Photobiomodulation Or biostimulation); (Osteoblasts proliferation AND differentiation); (Platelet-Rich Plasma OR PRP); (Platelet-Rich Fibrin OR PRF); (Growth Factor AND Concentrated Growth Factor OR CGF); (Mesenchimal Stem Cell AND Bone regeneration); (Bone morphogenic protein AND Bone regeneration); (Sinus flor elevation OR Sinus lift); (Alveolar Ridge Augmentation OR Socket Preservation); ((bone scaffolds OR bone graft OR bone substitutes) AND Hyaluronic acid) ((microbiota* OR microbiome *) AND boneregeneration))” Timespan: All years. Databases: PubMed/Medline, PubMed/Central, Web of Science and Google scholar

**Table 2:** Summary of the laser protocols for biomaterials osseointegration enhancement.

	Type of LLLT	Type of Irradiation	Groups Stydy	Results	Conclusion
Nagata et al. [208]	InGaAIP (λ 660 nm)	Power 35 mW/point Energy density 4.9 J/cm^2^/point	1-LLLT alone 2-(BMA) bone marrow aspirate 3-LLLT/BMA 4-control group with a blood clot	-Not suitable for proliferation of osteoblasts cell-Proliferation and differentiation was seen only for MSC present in BMA	The use of LLLT alone did not induce osteoblast proliferation but BMA/LLLT is a promising combined therapy in bone regeneration
Garcia et al. [209]	InGaAIP (λ 660 nm)	Power 35 mW/point energy density 4.9 J/cm^2^/point	1-control group with a blood clot 2-dexamethasone with a blood clot 3-dexamethasone+autologus bone 4-dexamethasone+LLLT 5-autologus bone+LLLT	-Dexamethasone group show less bone formation with a reduction in osteoblasts -Group treated with AB/LLLT osteogenic potential	LLLT helped bone from the inhibitory effects of dexamethasone LLLT improve bone healing in critical defects
Saygyn et al. [210]	Diode laser (λ 685 nm)	Power 25 mW/point energy density 2 J/cm^2^/point	1-MSC single dose irradiated 2-MSC double dose irradiated 3-control group	-Double dose group stimulate the release of IGFBP3, IGF-1 and bFGF-LLLT stimulate osteoblasts proliferation	LLLT improved wound healing and bone regeneration
Cunha et al. [211]	GaAIAs (λ 780 nm)	Power 100 mW/point energy density 6 J/cm^2^/point	1-LLLT group 2-autogenous bone 3-autogenous bone+LLLT 4-inorganic bovine bone 5-inorganic bovine bone+LLLT 6-contol group	-LLLT stimulates new bone formation	Laser accelerated graft material particles and bone healing
de Olivera et al. [212]	GaAlAs (λ 808 nm)	Power 100 mW/point energy density 4 J/cm^2^/session	1-LLLT major group 2-control major group (each major group divided in three groups-coagulum-inorganic bovine bone, -HA/TPC)	-LLLT group shows osteogenic potential -expression of BMP2, Osteocalcin, ALP and genes (Runx2, Jagged1) -Maintained the volume of biomaterials -Osteoblastic differentiation	LLLT stimulated bone formation in grafted area with osteoconductive materials
de Olivera et al. [213]	GaAlAs (λ 808 nm)	Power 100 mW/point energy density 4 J/cm^2^/session	1-deproteinized bovine bone (DBB) 2-HA/TCP 3-LLLT+DBB 4-LLLT+HA/TCP	-LLLT group osteogenic potential with the expression of BMP2 and OCN-increase of implant osteointegration	LLLT increased osteointegration in grafted area with osteoconductive materials
Gerbi et al. [214]	GaAlAs diode laser (λ 830 nm)	Power 40 m/W/point energy density 4 J/cm^2^/point	1-control group 2-LLLT group 3-BMP+membrane 4-BMP+membrane+LLLT group	-Osteogenic potential	LLLT combined with the use of biomaterials accelerated bone regeneration process
Renno et al. [215]	GaAlAs diode laser (λ 830)	Power 30 m/W energy density 10 J/cm^2^	1-MC3T3 grown on biosilicate+LLLT 2-control group	-LLLT GROUP 13% decrease in cell proliferation	LLLT group resulted in a reduction in cell growth
Grassi et al. [216]	Laser diode (λ 920 nm)	Power 0.1 W energy density 3 J/cm^2^	1-Osteoblasts-like cells seeded on zirconia or titanium surface+LLLT 2-control group	-Osteogenic potential -cell proliferation-cell differentiation-ALP expression -the mRNA of RUNX2 and OSTERIX	LLLT significantly increased cellular adhesion on implant surface
Pagin et al. [217]	Visible red (λ 660 nm) Infrared (λ 780 nm) LED (λ 630 ± 10 nm)	Laser: power 1 W/cm^2^ energy density 3 J and 5 J/cm^2^ LED: power 60 mW/cm^2^ energy 3 J and 5 J/cm^2^	MC3T3 irradiated with red/infrared laser and LED	Red/infrared and LED -influenced ALP -no effect on cell differentiation	Red/infrared laser and LED had similar effects et early periods of time on stimulating pre-osteoblasts
Queiroga et al. [218]	Red spectrum (λ 660 nm) infrared (λ 780 nm)	Power 40 mW energy density 2 J/point	1-LILT 660 nm 2-LILT 780 nm 3-Control group	-LILT with 780 nm newly formed bone -LILT with 660 nm no difference from control group	LILT with 780 nm wavelength promote bone reparation
Mergoni et al. [219]	Diode laser GaAs (λ 915 nm)	Power 0.12 and 1.25 W/cm^2^ 5.15 and 45 J/cm^2^	-Osteoblasts isolated from mandibular cortical LLLT treated -control group	-No osteoblast cell proliferation -no osteoblast cell differentiation	LLLT induced more bone nodules formation
Jawad et al. [220]	Diode laser GaAlAs (λ 940 nm)	Power+energy 100 mW/45.85 J/cm^2^ 200 mW/91.79 J/cm^2^ 3000 mW/137.57 J/cm^2^	-LLLT groups -Control group	-Cell proliferation -cell differentiation -ALP and osteocalcin expression	LLLT improved bone formation by stimulating osteoblast cells

**Table 3:** Summary of the biomaterials and graft for sinus augmentation procedure.

Sinus Augmentation Bone Substitutes		
Minetti et al. 2019 [132]	Demineralized and granulated tooth; Disinfected dental matrix	23 patients; 40 implants; ridge height 5.22 ± 2.04 mm increased to 14.72 ± 2.83 mmbone healing. At six months 1 implant fail; 97.5% survival rate
Lui et al. 2020 [304]	Deproteinized bovine bone delay implant placement (two-stage) MSFE; resorbable membrane	20 patients; 36 implants placement; The loss of bone volume: test 13.29 ± 8.56% vs. control 12.87 ± 5.15%; ISQ test group vs. 71.85 ± 5.59 increased to 80.42 ± 3.38 ISQ for the control group was 72.46 ± 4.86 increased to 82.39 ± 1.57
Younes et al. 2019 [305]	Deproteinized bovine bone After 4 months of implant placement	22 patients; 50 implants (2 weeks; 3 months; 2 years) Graft volumes amounted at 2 weeks 1418.26 mm^3^, at 3 months 1201.21 mm^3^ at 2 years 1130.13 mm^3^ graft volume stability of 79.7%.
Fouad et al. 2018 [306]	Demineralized bovine bone with simultaneous implant placement and collagen membrane	17 patients- 20 sinus lift; six months follow-up: Bone height was increase 8.59 ± 0.74 mm Bone density was 375.59 ± 49.38 ISQ values was 78.3 ± 5.08
Mazzocco et al. 2014 [307]	An organic bovine bone with a bioresorbable collagen membrane; simultaneous implant placement and delayed with nine months placements	20 patients; 8–9 months later control Graft volume amount: immediately after procedure (T1): 1.432 ± 539 mm^3^ 8–9 months later (T2): 1.287 ± 498 mm^3^ Graft volume contraction from T1 to T2 was 10%
Younes et al. 2016 [308]	Bovine-derived bone and collagen membrane implant placement after 4.6 ± 1.5 months	57 patients; 53 sinus lift; 105 implants placement Implant survival was 99% after 19 ± 9 months. Bone height at the beginning was 3.87 ± 1.74 mm Bone height at the moment of implantation and final control: 13.7 ± 2.12 mm and 13.11 ± 2.12 mm ICC for marginal bone loss was 0.96 (*p* < 0.001)
Scarano et al. 2017 [309]	Decellularized bovine compact bone, collagen membrane and implant placement after six months	4 patients; six months control Graft bone volume Immediately postoperative was 2106 mm^3^ After 6 months was 2053 mm^3^
Olaechea et al. 2019 [310]	Biphasic HA/β-TCP-30/70% bony closed with a collagen membrane	10 patients; six month control increase in vertical bone height 8.03 ± 1.72 mm mineralized tissue 34.93 ± 14.68% non-mineralized tissue 55.23 ± 11.03% remnant biomaterial 9.82 ± 11.42%
Olaechea et al. 2016 [311]	Β-TCP and simultaneous implant placement	30 patients; 58 implants Bone volume decrease: immediately after surgery: 1206.9 ± 437 cm^3^ 6 months after surgery: 912.6 ± 356 cm^3^ 2.5 years after surgery: 662 ± 294 cm^3^ 2.5 years after surgery, 41/58 implants were without bone around the tip of the implant
Oba et al. 2020 [312]	Β-TCP bone graft with immediate implant placement	23 patients; 30 implants placement; ≥3 years follow-up Height of the augmented sinus floor: from 6.54 ± 1.51 to 3.11 ± 1.35 mm Height of the bone above the implant apex: from 3.17 ± 0.97 to −0.25 ± 1.19 mm
Ohe et al. 2016 [313]	Biphasic calcium phosphate (BCP), collagen membrane and implant placement in one stage	15 patients; 16 sinus lift Bone graft volume decreased to 1117.04 ± 686.74 mm^3^ from 1350.44 ± 562.56 mm^3^ Graft maintained 82.71% until post-op 6 the average volume loss is 203.73 mm^3^ (about 0.20 cc)

**Table 4:** Summary of the biomaterials and graftfor ridge preservation procedure. [T: Test; C; Control].

Ridge Preservation Bone Substitutes		
Minetti et al. 2019 [131]	Demineralized and granulated autologous tooth graft; collagen membrane	98 patients; 119 socket sites; 106 implantations; 4-month implant placement; follow-up 9–45 months The mesio-distal defect was 10.3 mm, buccal lateral/palatal 7.0 mm, and vertical 9.16 mm. After 4 months: bone volume was 41.47 ± 11.51%; residual graft was 16.60 ± 7.09%; vital bone was 21.89 ± 9.72%
Valdec et al. 2017 [319]	Demineralized autologous tooth graft	4 patients; 3–4 months implant placement 1 year after prosthetic procedure: vertical dimension: a loss of 0.76 mm horizontal dimension: a loss of 1.1 mm
Minetti et al. 2020 [320]	Demineralized deciduous teeth material	1 patient; 2 alveolar socket grafted; 2 years follow-up2 implant placement after 4 months (3.1, and 4.1). Bone volume 47.22%. residual graft volume 18.68%, vital bone 28.55%
Del Canto-Diaz et al. 2018 [321]	Autologous dental material Collagen membrane	6 patients; follow-up 8–16 weeks; Bone lost: Vertical from bottom to lingual crest (VL): control group 1.77 mm, autologous tooth-derived graft material group 0.42 mm. Height differences from lingual to buccal cortical bone decrease (HL-BCB): control 2.22 mm Autologous Tooth-Derived Graft Material 0.16 mm
Al Qabbani et al. 2018 [322]	Lyophilized bovine bone and resorbable membrane	20 patients; followed up until 9 months Comparisons within the groups showed a significant difference in bone resorption between the two groups: 1.49 mm at 3 months in the grafted group 1.84 mm at 9 months in the control group
Fischer et al. 2018 [323]	T1-demineralized bovine bone/soft tissue punch T2-demineralized bovine bone T3-demineralized bovine bone/collagen membrane T4-control group non treated	35 patients; 35 single-gap extraction sites; 6 months implant placement and control. Bone resorption at each group after 6 months: T1 −0.874 ± 0.713 mm T2 −0.968 ± 0.344 mm T3 −1.26 ± 0.942 mm T4 −2.15 ± 1.349 mm Bovine bone/control group (>1 mm/<2 mm)
Pang et al. 2014 [324]	Deproteinized bovine bone and collagen membrane, delay implant placement after 6 months	30 patients; 6 months Bone height: Test 1.54 (0.25) mm Control 3.26 (0.29) mm; Bone width: Test 1.84 (0.35) mm Control 3.56 (0.28) mm Bone volume Test 262.06 (33.08) mm Control 342.32 (36.41) mm
Naenni et al. 2018 [325]	T1-(PLGA): 60%/40% HA/ß-TCP and collagen membrane T2-biphasic calcium phosphate 60%/40% HA/ß-TCP and collagen membrane T3-control group	16 dogs experiment; 62 extraction site Pre-extraction to sacrifice: Median buccal volume change: T1: −1.76 mm T2: −1.62 mm T3: −2.42 mm Ridge width change: T1: −2.51 mm T2: −2.04 mm T3: −3.85 mm
Ikawa et al. 2016 [326]	TG: β-TCP block (TCP, polyvinyl alcohol, distilled water) CG: no graft	6 dogs-animal experiment; Bone loss measurements: Coronal/middle horizontal width: TG: 3.2 ± 0.5 mm/3.6 ± 0.4 mm CG: 1.2 ± 0.3 mm/2.0 ± 0.6 mm Amount of woven bone: TG: 62.4% ± 7.9% CG: 26.8% ± 5.3% Connective tissue and bone marrow: TG: 10.7% ± 5.7%/4.1% ± 2.2% CG: 38.1% ± 6.2%/16.0% ± 6.9%
Mayer et al. 2016 [327]	test group (T)—composite BCS/BCP, (Biphasic calcium sulphate with β Tri-Calcium Phosphate and Hydroxyapatite) control group (C)—no grafting material	36 patients; 40 extraction sockets; 29 follow-up Horizontal ridge width change 4 months (T) at −3 mm from crest: 0.03 ± 2.32 mm (C) at −3 mm from crest: 2.28 ± 2.36 mm (T) at −6 mm from crest: 0.035 ± 3.05 mm (C) at −6 mm from crest: 2.28 ± 2.43 mm Vertical ridge change 4 months (T) 0.307 ± 2.01 mm (C) 0.14 ± 2.03 mm Total bone/connective tissue/residual graft (T) 47.7 ± 10.6/36.3 ± 19.4/15.9 ± 11.4 (C) 52.6 ± 11.6/46.7 ± 10.6/NA
Baranes et al. 2019 [315]	Biphasic calcium sulfate	Composite biomaterial for small osseous defects and extraction sockets

**Table 5:** Comparative summary of the main biomaterials’ properties.

	Ideal/Autologous Graft	TCP	Dentine Matrix	Bovine Bone
Biocompactibility	++	++	++	++
Mechanicalproperties	++	+ -	++	++
Osteogenic	++	- -	+ -	- -
Osteoconductivity	++	++	++	++
Osteoinductivity	++	- -	+ -	- -
Resorbtion	Regular	Fast resorbtion	Slow resorbtion	Slow resorbtion

**Table 6:** Comparative summary of the healing characteristics of the bone substitutes (AB: autogenous bone; AP: alloplastic graft; XG: xenografts; DM: dentin matrix; NBR: New Bone Regeneration; RG: Residual Grafts; CT: Connective Tissue).

	NBR	RG%	CT%
Healing time < 6 months	AB > AP	AB > AP	AB > AP
	AB > XG	AB < XG	AB > XG
	AB > DM	AB < DM	AB > DM
Healing time ≥ 6 months	AB > AP	AB > AP	AB > AP
	AB ≈ XG	AB < XG	AB > XG
	AB ≈ DM	AB < DM	AB ≈ DM

## Data Availability

All experimental data to support the findings of this study are available by contacting the corresponding author upon request. The authors have annotated the entire data building process and empirical techniques presented in the paper.

## Abbreviations

3Ca_3_(PO_4_)_2_Ca(OH)_2_	hydroxyapatitepurum
3D	three-dimensional
ADM	autologous tooth-derived graft material
Alb-CGF	autologous albumin gel and concentrated growth factor
Alb-PRF	autologous albumin gel and platelet-rich fibrin
ALP	alkalin phosphatase
Ang4	angiogenin-4
APAG	activated plasma albumin gel
APDDM	autogenous partially demineralized dentin matrix
A-PRF	advanced platelet-rich fibrin
AT	adipose tissue
ATP	adenosine triphosphate
AT-SCs	adipose tissue-stem cells
BCP	biphasic calcium phosphate
bFGF	basic fibroblast growth factor
BHA	bone hydroxyapatite
BM	bone marrow
BMA	bone marrow aspirate
BMPs	bone morphogenetic proteins
BM-SCs	bone marrow-stem cells
BRONJ	bisphosphonate-related osteonecrosis of the jaw
Ca	calcium
Ca^2+^	calcium ion
Ca_2_P_2_O_7_	calcium pyrophosphate
Ca(OH)_2_	calcium hydroxide
CBB	compact bio-bone
CD34	encoded gene in human and other species
CG	control group
CGF^®^	concentrated growth factor
CNS	central nervous system
CO_3_(^2−^)	carbonate ion
CS	calcium sulphate
DBB	deproteinized bovine bone
DC	dendritic cells
DDM	demineralized dentin matrix
DMP	gene protein coding (dentin matrix acidic phosphoprotein)
E2	estradiol
EGF	epidermal growth factor
EGFR/Akt/PI3K	epidermal growth factor receptor/protein kinase B/phosphatidylinositol-3-kinase
EMD	enamel matrix protein derived
ENS	enteric nervous system
FA	amniotic fluid
FA-MSCs	amniotic fluid-stem cells
fMWCNT	functionalized multi-walled carbon nanotube
GBR	guided bone regeneration
GCT 30	gelatin-chitosan-β-TCP 30%
GF	germ free
GF	growth factor
HA	hydroxyapatite (Ca_10_(PO_4_)_6_(OH)_2_)
Ha	hyaluronic acid
HA/TCP	combination of hydroxyapatite with tricalcium phosphate
HL-BCB	vertical measurements from buccal cortical bone
hMSC	human mesenchymal stem cells
IFN	interferon
IFNγ	interferon gamma
IGF	insulin-like growth factor
IGFBP3	insulin-like growth factor-binding protein 3
IL-1	interleukin-1
ITAM	immunoreceptor tyrosine-based activation motif
LED	light-emitting diode
LLLT	low-level laser therapy
L-PRF	leukocyte platelet-rich fibrin
MCS	mesenchymal stem cell
MDM	mineralized dentin matrix
MMPs	matrix metalloproteinases
MRONJ	medication-related osteonecrosis of the jaw
MSFE	maxillary sinus floor elevation
Na+	sodium ion
NHA	nano-hydroxyapatite
NK	natural killer
Oct4	octamer-blinding transcription factor 4
ONC	osteocalcin
OPG	osteoprotegerin
P	phosphorus
PB	peripheral blood
PBM	photobiomodulation
PB-SCs	peripheral-blood stem cells
PCBM	particulate cancellous bone and marrow
PDGF	platelet-derived growth factor
PDGF	platelet-derived growth factor
PDGF-BB	platelet-derived growth factor-BB
Pl	placenta
PLGA	poly(lactic-co-glycolic acid)
Pl-SCs	placenta-stem cells
PPP	platelet-poor plasma
PRF^®^	platelet-rich fibrin
PRP^®^	platelet-rich plasma
R.P.M	revolution per minute
RANK	receptor activator of nuclear factor kappa-B
RANKL	receptor activator of nuclear factor kappa-B ligand
RBC	red blood cells
rhBMP/Bio-Oss^®^	bovine bone as a carrier of recombinant human bone morphogenetic protein
RT PCR	reverse transcription polymerase chain reaction
SCFA	short chain fatty acids
SCs	stem cells
SEM	scanning electron microscope
SFM	serum-free media
Sox2	sry-box-containing gene 2
SRP	scaling and root planing
SSEA-3	stage-specific embryonic antigen 3
T α/β + e γ/δ +	cell population
T1	tested group 1
TCP	tricalcium phosphate
TG	test group
TGF	transforming growth factor
TGF-β1	transforming growth factor-beta1
TNF	tumor necrosis factor
TT^®^	tooth transformer
UCB	umbilical cord blood
UCB-SCs	umbilical cord blood
VDR	vitamin D receptor
VEGF	vascular endothelial growth factor
VL	vertical line, tooth’s axis
VL-BCB	horizontal measurements from buccal cortical bone
WALT	world association for laser therapy
α-TCP	alfa-tricalcium phosphate
β-TCP	beta-tricalcium phosphate
β-TGF	beta-transforming factor
